# Novel approach to a severe craniomaxillofacial trauma in a military working dog using a customized 3D-printed mold for printed polymethylmethacrylate (PMMA) plate–a case report

**DOI:** 10.3389/fvets.2025.1647992

**Published:** 2025-12-15

**Authors:** Tatiana Babenko, Itay Srugo, Dana Peery, Efrat Kelmer, SIgal Klainbart

**Affiliations:** 1 Department of Neurology, The Veterinary Teaching Hospital, Koret School of Veterinary Medicine, The Robert H. Smith Faculty of Agriculture, Food and Environment, Hebrew University of Jerusalem, Jerusalem, Israel; 2 Department of Diagnostic Imaging, The Veterinary Teaching Hospital, Koret School of Veterinary Medicine, The Robert H. Smith Faculty of Agriculture, Food and Environment, Hebrew University of Jerusalem, Jerusalem, Israel; 3 Department of Small Animal Emergency and Critical Care, The Veterinary Teaching Hospital, Koret School of Veterinary Medicine, The Robert H. Smith Faculty of Agriculture, Food and Environment, Hebrew University of Jerusalem, Jerusalem, Israel

**Keywords:** frontal bone, reconstruction, head trauma, facial deformation, cribriform plate, pneumocephalus, frontal sinus

## Abstract

**Objective:**

To describe a novel two-stage surgical approach for managing complex craniomaxillofacial trauma in a military working dog, including the use of an individualized 3D-printed polymethylmethacrylate (PMMA) plate for frontal bone reconstruction.

**Case summary:**

A 3-year-old Belgian Malinois sustained severe head trauma during combat. Clinical examination revealed facial deformation, respiratory distress, and neurological signs. computerized tomography (CT) imaging confirmed multiple fractures, including the frontal and nasal bones, cribriform plate, and frontal sinus, with pneumocephalus. A two-stage approach was employed: (1) Initial stabilization, including a tracheostomy to address respiratory issues and manage intracranial pressure, along with surgical debridement and closure of the frontal sinus. (2) Reconstruction using an individualized 3D-PMMA plate using a customized 3D-printed mold to restore frontal bone integrity.

**New or unique information provided:**

This report presents a novel approach to managing complex craniomaxillofacial trauma in dogs, particularly those involving extensive frontal sinus and nasal bone fractures with intracranial complications. The use of an individualized 3D- PMMA plate for frontal bone reconstruction represents a significant advancement in veterinary CMF trauma management, offering a potential solution for achieving both functional and cosmetic outcomes in challenging cases. This case contributes to the limited existing literature on the management of severe frontal sinus fractures in dogs.

## Introduction

Craniomaxillofacial (CMF) trauma in dogs presents a complex challenge, often involving multiple anatomical structures and necessitating individualized treatment strategies to achieve optimal recovery outcomes. Published veterinary studies have examined CMF fracture characteristics, including location, morphology, classification, etiology, and demographic data (1, 2) and the associations between these factors and prognosis (2–4). Facial fractures combined with cranial vault fractures–affecting bones such as the frontal, parietal, temporal, occipital, or ethmoidal–account for ~33% of all skull fractures in dogs, with frontal bone fractures representing 17% of cases (1). In humans, frontal sinus fractures are classified as anterior or posterior wall fractures with or without displacement and cerebrospinal fluid (CSF) leakage (5). Almost all posterior fractures are treated surgically because they can create a pathological connection between the frontal sinus and the cerebral cavity, potentially leading to life-threatening complications (6). A critical knowledge gap exists regarding the treatment of anterior frontal sinus fractures in dogs. To date, only a limited number of case reports have been published (4, 7–10), and no established treatment guidelines exist. Furthermore, no cases have been reported to date of the treatment of complex posterior and anterior frontal sinus fractures together with nasal bone and cribriform plate fractures, leading to CSF leakage, brain parenchyma trauma, and compression, along with pneumocephalus.

This case report describes a novel two-stage surgical approach for managing complex CMF trauma in a military working dog alongside intensive care. The treatment included the use of an individualized polymethylmethacrylate (PMMA) plate, prepared during surgery using a customized 3D-printed mold for frontal bone reconstruction, highlighting a potential advancement in veterinary CMF trauma management.

## Case summary

**Figure 1:**
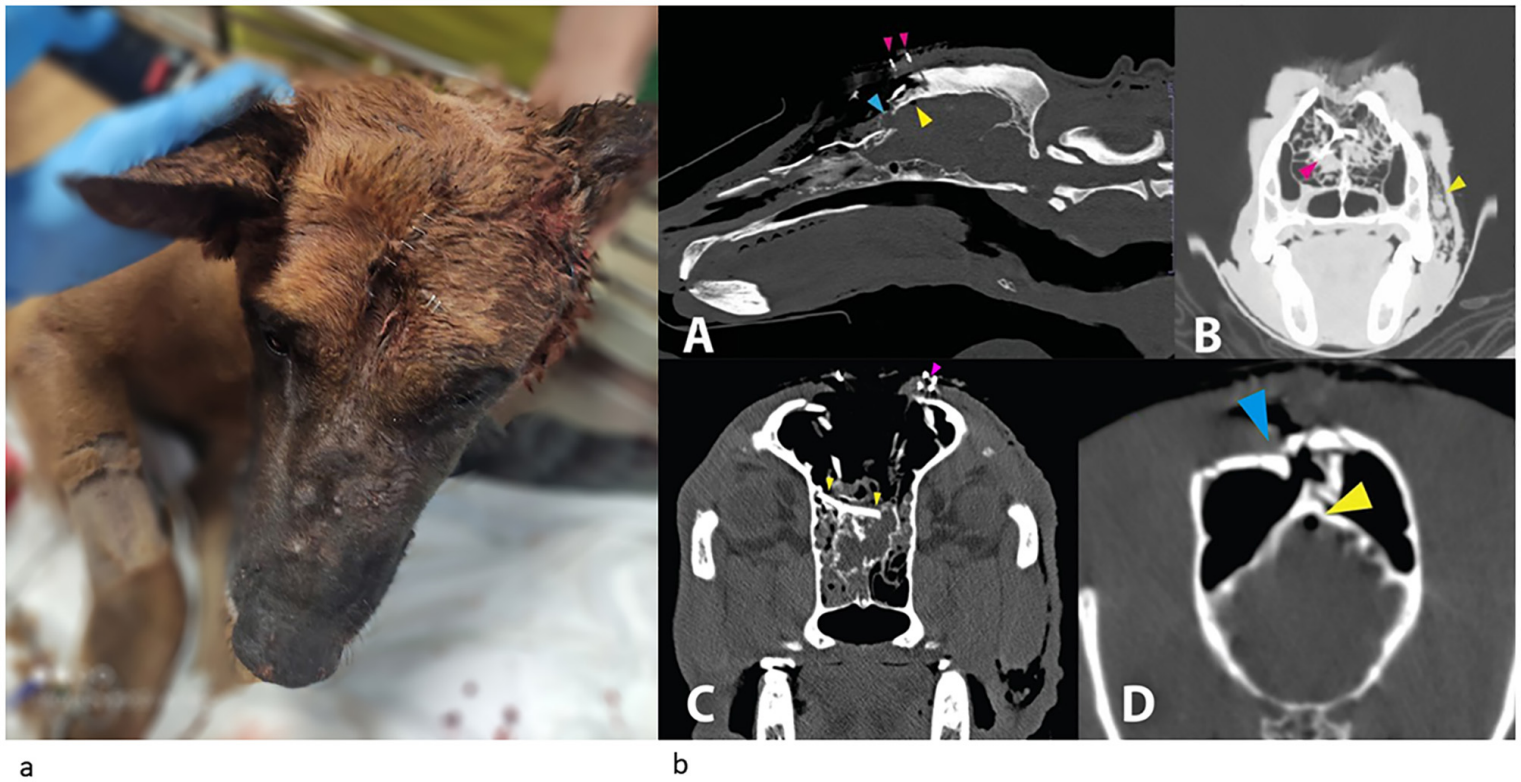
**(a)** Photograph of the patient upon admission. Note the facial deformation with frontal bone depression. Some skin and soft tissue wounds were closed with staples, while those over the area of frontal sinus wall depression were left open to prevent breathing difficulties. **(b; A)** Mid sagittal image in a bone window showing multiple rostral skull fractures, including a depressed fracture of the nasal part of the frontal bone (blue arrowhead), allowing direct communication between the frontal sinuses and brain. A small amount of gas is seen ventral to the frontal bone within the calvarium indicating pneumocephalus (yellow arrowhead). Multiple nasal and additional frontal bone fractures are also seen with complete breaching of the frontal sinuses. Serial small metal opacities represent surgical clips (pink arrowheads). **(B)** Transverse, lung window view at the level of the maxillary recesses. Fractures of the lacrimal, frontal and nasal bones are apparent. A large bone fragment is depressed into the nasal cavity (pink arrowhead). Destruction of nasal turbinates and areas of increased soft tissue opacity, likely representing hemorrhage are evident. [Sub-cutaneous emphysema (yellow arrow head) is also noted]. **(C)** Transverse view at the level of the zygomatic arch and frontal sinuses demonstrating multiple fractures of the frontal and ethmoidal bones also involving the cribriform plate. A large bone fragment (yellow arrowheads) depressed into the rostral brain parenchyma. The sinus wall has collapsed with multiple small bone fragments demonstrated within the sinuses. Pink arrowhead denotes metallic surgical clips. Sub cutaneous emphysema is also apparent. **(D)** Transverse image at the level of the mandibular ramus showing comminuted depressed frontal bone fractures and breaching of the frontal sinus walls (bleu arrowheads). A focal small gas opacity region within the cranial vault is compatible with pneumocephalus (yellow arrowheads).

A 3-year-old, 34 kg, Belgian Malinois military attack dog, was presented to the emergency department of a Veterinary Teaching Hospital 6 h after sustaining a severe head injury from blunt and sharp force trauma (a hammer and a knife) during a combat incident. The dog was treated in the field with 1.5 Liters of Lactated Ringer solution IV, 500 mg tranexamic acid IV, 1 g cefalexin IV, 300 mg enrofloxacin IV, and 100 mg pethidine SQ. On physical examination upon admission, the dog was sedated and nonambulatory. Heart rate was 140 beats per minute, Respiratory rate was 20 beats per minute, and the rectal temperature was 38.6 °C. The dog had strong, synchronous peripheral pulses, with pink mucous membranes, CRT < 2 s, no cardiac murmurs or arrhythmias, and normal bilaterally bronchovesicular sounds. Body condition score was 5/9. Severe facial deformation with frontal bone depression was noticed on the projection of the frontal sinuses bilaterally, extending from the bregma landmark to the caudal edge of the nasal bones at the level of the medial canthus of the eyes, with palpable crepitations and unstable bone fragments. Several skin and soft tissue wounds were present above the site of the bone depression (Figure 1a), with pathological movement of the skin in coordination with breathing, and airflow through the wounds. Modified Glasgow Coma Scale score was 15/18, no cranial or spinal nerve deficits were noted, and there were voluntary movements of all four limbs. Normal ambulation was reported by the canine unit's veterinarian during a physical examination before sedation and transport to the hospital. CBC^1^ and serum biochemistry^2^ panel were unremarkable, except for moderate hypoalbuminemia and hypoproteinemia [2.7 g/dl; reference interval (RI), 3–4.4; 5.23gr/dl; RI, 5.4–7.6; respectively], mild elevation of muscle enzymes (creatine phosphokinase, 3,063 IU/L; RI, 51–399; Aspartate aminotransferase, 131 IU/L; RI, 19–42) and mild hypokalemia (3.32 mmol/L; RI, 3.7–5.4). Abdominal and thoracic point-of-care ultrasound scans were unremarkable. The wounds were clipped and cleaned with sterile isotonic solution (chlorhexidine and sodium chloride, 0.9%).^3^^,^^4^ An attempt to temporarily close the skin wounds with staples to avoid intracranial contamination was made, however, this caused breathing difficulties, necessitating the removal of the staples. The dog received an intravenous bolus of fentanyl^5^ (3 μg/kg) succeeded by a constant rate infusion (CRI) at 5 μg/kg/h, IV, Lactate ringer solution^6^ 2.5 ml/kg/h, and clindamycine^7^ 12.5 mg/kg, IV. Following initial stabilization, a non-contrast computed tomographic (CT) scan of the head and thorax was performed while the dog was sedated with fentanyl 5 μg/kg/h IV, midazolam^8^ 0.4 mg/kg, IV, and propofol^9^ 1 mg/kg, SIV. The scan revealed numerous skull fractures involving the face and cranial vault, including multiple open comminuted and depressed nasal, maxillary, ethmoidal, and frontal bone fractures. Complete breaching of the frontal sinus walls, septa, and the overlaying soft tissues, and destruction of nasal structures with severe accompanying facial subcutaneous emphysema were apparent. Multiple fractures of the cribriform plate were noted, with a large frontal bone fragment depressed into the rostral brain parenchyma. A focal small hyperattenuating region within the left olfactory lobe was presumed to be a hemorrhage. More caudally, a small area of intracranial gas opacity, compatible with mild pneumocephalus, was seen (Figure 1b).

Considering the pathological airflow through the frontal bone defect into the skull cavity, the compression of the brain parenchyma by a cribriform plate fragment during breathing, and the suspected sinonasal obstruction blocking normal upper respiratory pathways, a temporary tracheostomy was performed to bypass the upper respiratory pathways, preventing additional parenchymal compression from the cribriform plate fragments and progression of pneumocephalus. The dog was hospitalized in the ICU and was treated with Lactate ringer solution, 2.5 ml/kg/h with added metoclopramide^10^ 1 mg/kg/24 h, fentanyl/lidocaine^11^/ketamine,^12^ 3 μg/kg/h, 2, 0.2 mg/kg/h, respectively; clindamycin, 12.5 mg/kg, IV, q12h; enrofloxacin,^13^ 10 mg/kg, IV q24h; levetiracetam,^14^ 20 mg/kg, IV, q8h; Maropitant,^15^ 1 mg/kg, SIV, q24h; pantoprazole,^16^ 1 mg/kg, IV q12h, and Trazadone,^17^ 3 mg/kg, PO/PR as needed.

**Figure 2:**
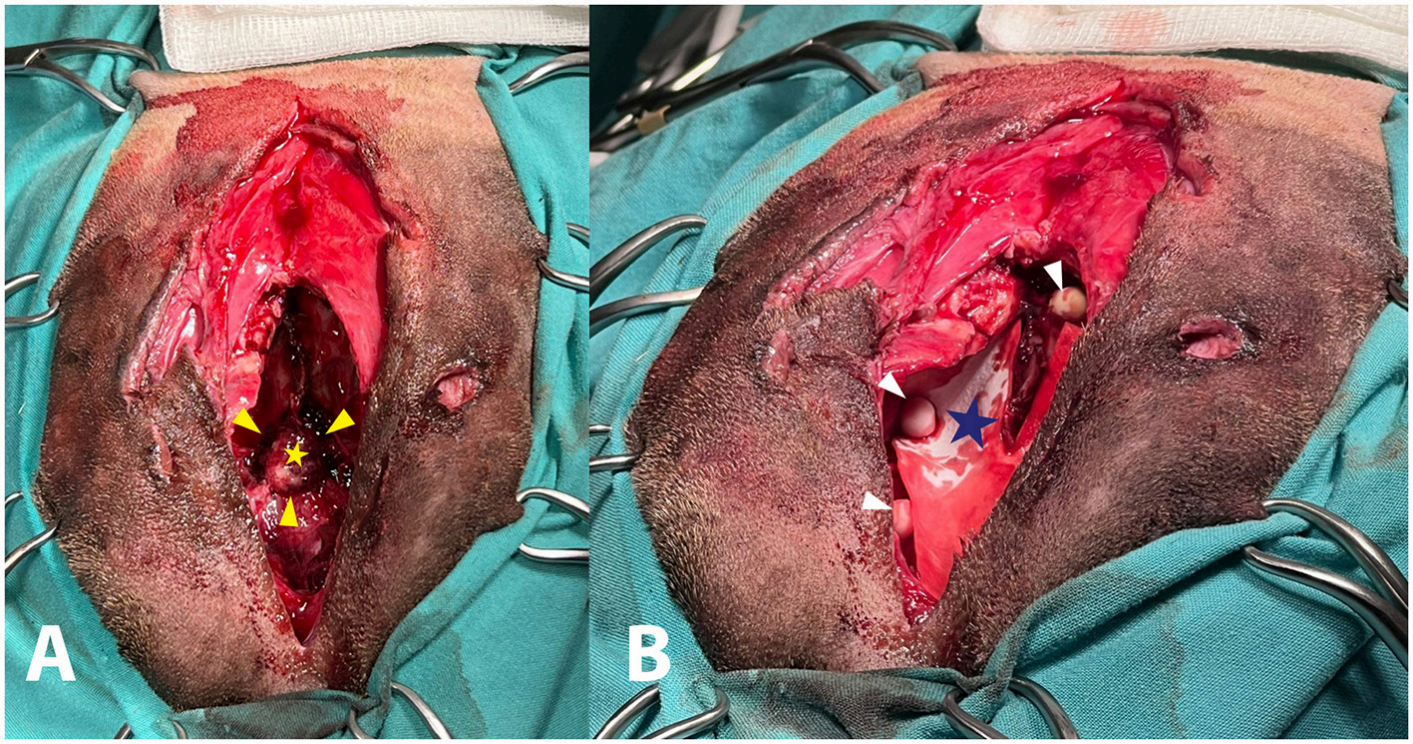
**(A)** A view of the wound in the frontal sinus after the removal of bone fragments and flushing revealed a hole in the internal frontal bone (yellow arrows) and the olfactory lobe parenchyma (yellow star) visualized in depth. **(B)** A view of the wound in the frontal sinus after the placement Dural Regeneration Matrix **(A)** (blue star), and restorable granules with antibiotics Amikacin and Meropenem (white arrows).

**Figure 3:**
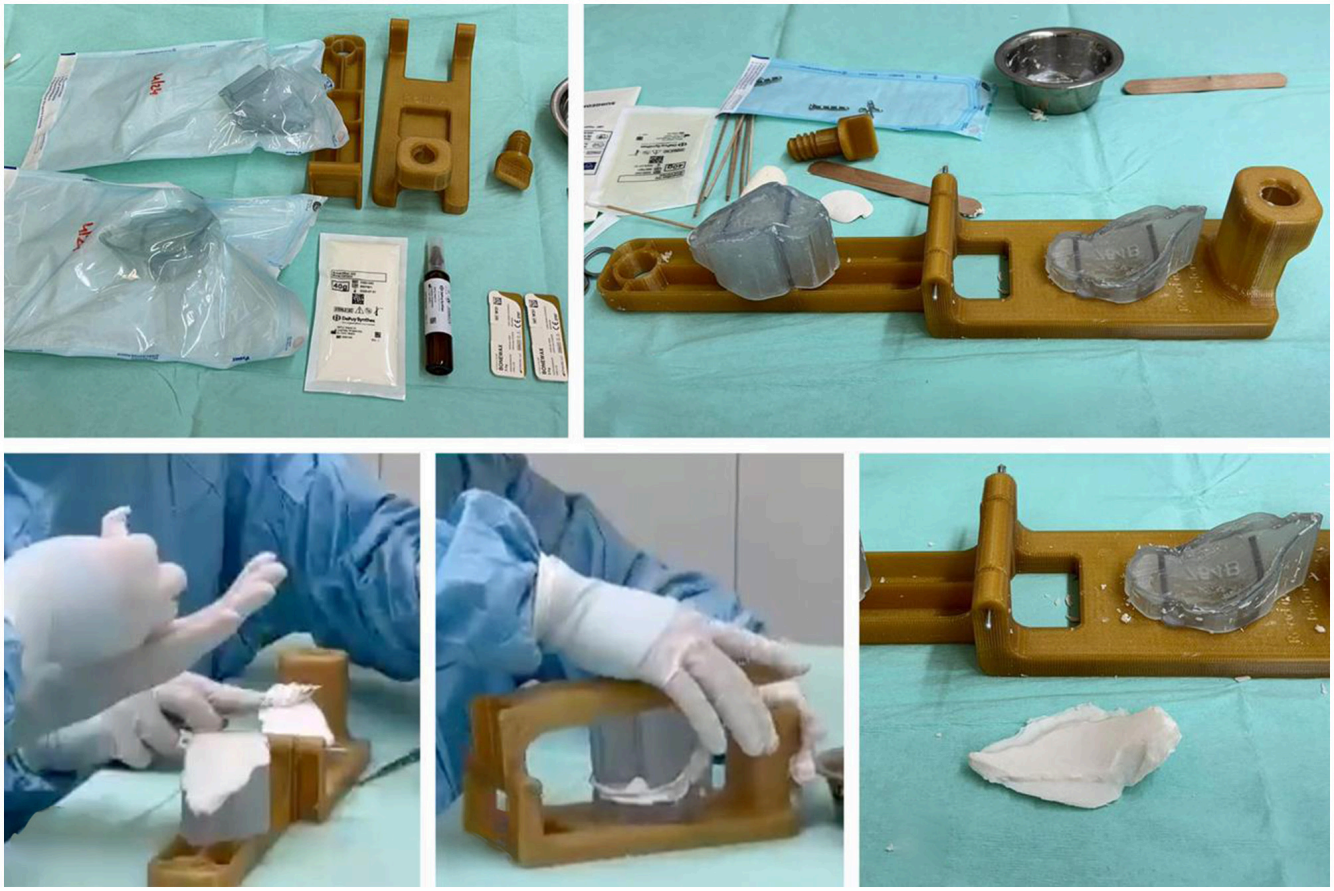
Preparing of the individualized patient-specific 3D PMMA implant during surgery, using 3D-printed cast molding device, made from two separate individual 3D-printed parts, placed on clamp. Before placing PMMA, the surface of each part of cast molding device was covered by thing lay of Bonewax (Ethicon) for facilitate removal of PMMA implant after hardening.

The first surgery was performed the following day. Pre-medication included Fentanyl-lidocaine-ketamine, induction with propofol 0.6 mg/kg + diazepam,^18^ 0.05 mg/kg, IV, and maintained with isuflorane.^19^ The dog was placed in ventral recumbency elevated head and secured to the surgery table. A midline skin incision was made with a number 10 scalpel blade and extended from the bregma landmark to 30 mm rostral frontonasal junction. In the central part of the incision, where multiple skin wounds were located, the incision was made with a 5 mm lateral indentation on both sides of the most lateral wounds. Access to the free and non-free bone fragments and frontal sinus cavities was obtained immediately afterward. The muscles covering the frontal and caudal aspect of the nasal bones (frontalis, interscutularis, frontoscutularis, and the proximal part of the levator nasolabialis) were incised along the midline and retracted axially to provide better access to the entire frontal sinus. All free bone fragments and visible pieces of gravel were removed. The surgical site and sinus cavity were flushed with large volumes of sterile 0.9% saline solution to remove residual sand, gravel, small bone fragments, and blood clots. Visualization of all areas was achieved, providing access to the hole in the internal frontal bone, olfactory lobe parenchyma, and the sinonasal junction (Figure 2A). A bacteriological swab culture was taken (which later yielded negative culture results), followed by the application of Oxidized regenerated Cellulose^20^ (Equitamp^®^) and Dural regeneration Matrix^21^ fixed by GLUture Topical Tissue Adhesive^22^ to replace the lost dura matter. These materials formed a hermetic seal over the hole in the internal frontal bone due to their adhesive layer. Intraoperative confirmation of hermetic seal was based on visual inspection and the absence CSF leakage upon a Valsalva maneuver (gentle positive pressure ventilation). The same implants were used to isolate the nasal cavity from the frontal sinus. Restorable granules with antibiotics (Amikacin and Meropenem granules, 9 mg in each granule)^23^ were placed throughout the frontal sinus and covered with sterile cellulose sponges^24^ (Figure 2B). After removing the damaged skin and smoothing the edges, the soft tissues were closed with simple interrupted sutures using 3-0 polydioxanone,^25^ and the skin was closed with simple interrupted sutures using 3-0 polyamide.^26^ The integrity of the frontal sinus and skull cavity was restored, but a large portion of the external frontal bone was lost. This resulted in a major bone defect and prevented a return to training due to the vulnerability of the frontal sinus to external damage. Postoperatively, the dog was hospitalized in the ICU. The tracheostomy tube was removed without complications 6 days after surgery. On the 7th day the dog was discharged with instructions for cage rest, clindamycin^27^ 12.5 mg/kg, PO, q12h, ofloxacin,^28^ 10 mg/kg, PO, q24h, and dipyrone,^29^ 25 mg/kg PO, q12h, all for 7 days. No infectious complications or CSF leakage were observed during the 4-week follow-up. A repeated CT scan was performed 3 weeks after surgery to assess the extent of bone loss and to create a model for replacing the bone defects. The DICOM files were exported to a CD, a commercial software package was used to simulate the cranioplasty,^30^ with an offset of 0.2 mm. The medical planner used the medical image as a base for the dog skull, the hole in the skull was made using a CAD software.^31^ An individualized 3D-printed PMMA plate was fabricated using a custom casting mold^32^ created by a 3D printer (see endnote v) and sterilized by steam autoclave, to replace the external frontal bone and prevent further brain trauma (Figure 3). The mold (Master-Clamp) consisted of two main components: a top part and a bottom part, connected by a 2.5 mm K-wire axis used to secure the mold sections. In addition, a closing screw was used to tighten the mold during preparation of the plate in surgery. During the procedure, the patient-specific mold was positioned on each part of the Master-Clamp. The inner surface of each mold was then coated with BONEWAX by applying a thick layer on both sides (two packs per side) to facilitate easy removal of the plate once hardened. PMMA was prepared in the standard manner, and the mixture was placed into the lower part of the mold using a spatula. The mold was then closed and secured with the screw, and any excess PMMA around the mold was removed using a curette. After 13 min, the mold was opened and the implant was removed.

**Figure 4:**
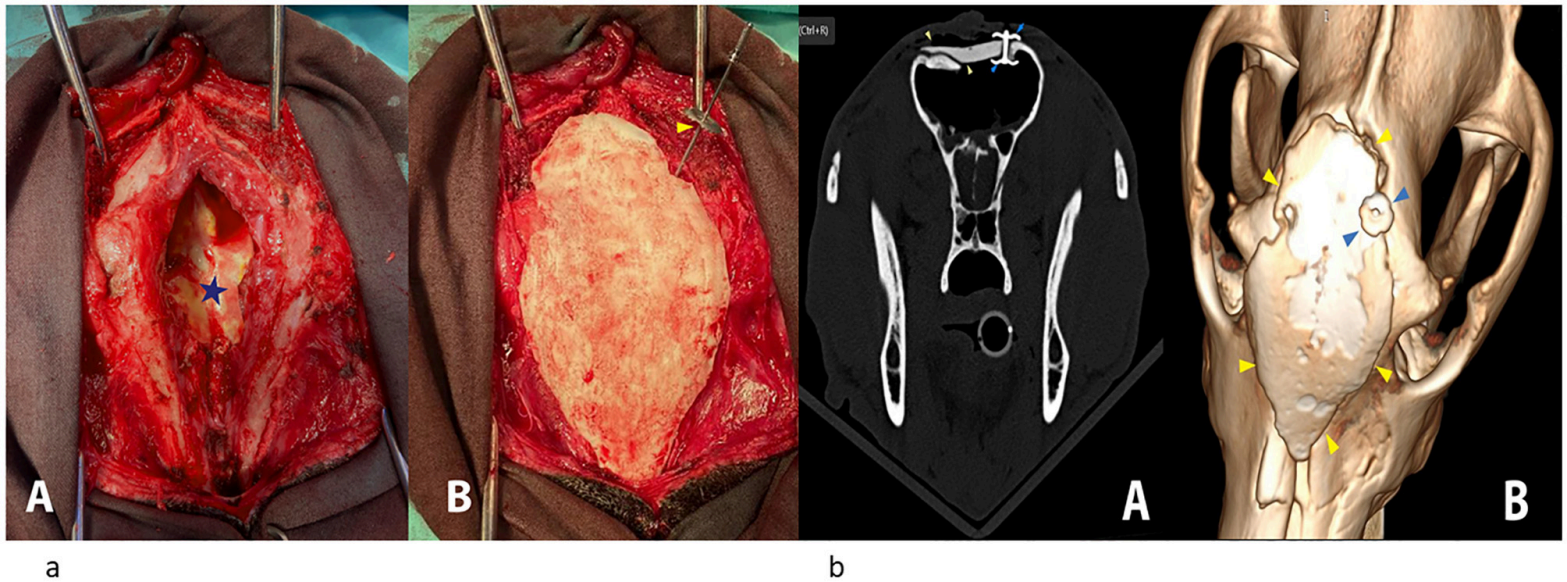
**(a; A)** A view of the wound in the frontal sinus after removal of skin and soft tissue scars and preparation of the frontal and nasal bone for plate placement. At the bottom of the sinus Dural Regeneration Matrix (DuraGen Secure) can be seen (blue star). **(B)** A view of the wound after placement 3D PMMA plate. The Aesculap^®^ CranioFix^®^2 system can be seen on the right dorsal side (yellow arrow). **(b)** Immediate post-surgical plating CT images. **(A)** Transverse bone window image showing bridging of the frontal sinuses wall gap by the 3D PMMA plate (yellow arrowheads). The blue arrowhead denotes a metal implant–Aesculap^®^ CranioFix^®^2 system used to anchor the plate and the frontal bone. **(B)** 3D reformatted dorsal view of the skull showing the 3D PMMA plate (yellow arrowheads). Blue arrows point at the Aesculap^®^ CranioFix^®^2 system.

The second surgery was performed 7 weeks following the first surgery. The dog was pre-medicated with methadone,^33^ 0.05 mg/kg + Acepromazine,^34^ 0.02 mg/kg, IM, induced by propofol, 0.3 mg/kg + diazepam, 0.05 mg/kg, IV, and maintained with isoflurane and placed in a ventral recumbent position with an elevated head and fixated to the surgery table. A skin incision was made with a number 22 scalpel blade around the scar from the previous surgery, extending 20 mm above and below. The underlying soft tissue scar was also incised to expose 30 mm of the bone around the hole in the external frontal bone and the caudal nasal bones. Access to the frontal sinus was achieved and maintained with two Gelpi retractors (Figure 4A). The sinus cavity was flushed with large volumes of sterile 0.9% saline solution. Meanwhile, the individualized 3D PMMA plate was prepared and the glue- GLUture Topical Tissue Adhesive^35^ (E) was applied to the periphery of the bottom of the plate, which would be adjacent to the dorsal surface of the bone. The plate was then placed in its position and fixed with the Aesculap^®^ CranioFix^®^2 system^36^ (Figure 4B). The soft tissues were closed with a simple Polydioxanone suture, followed by a simple skin Polyamide suture.

**Figure 5:**
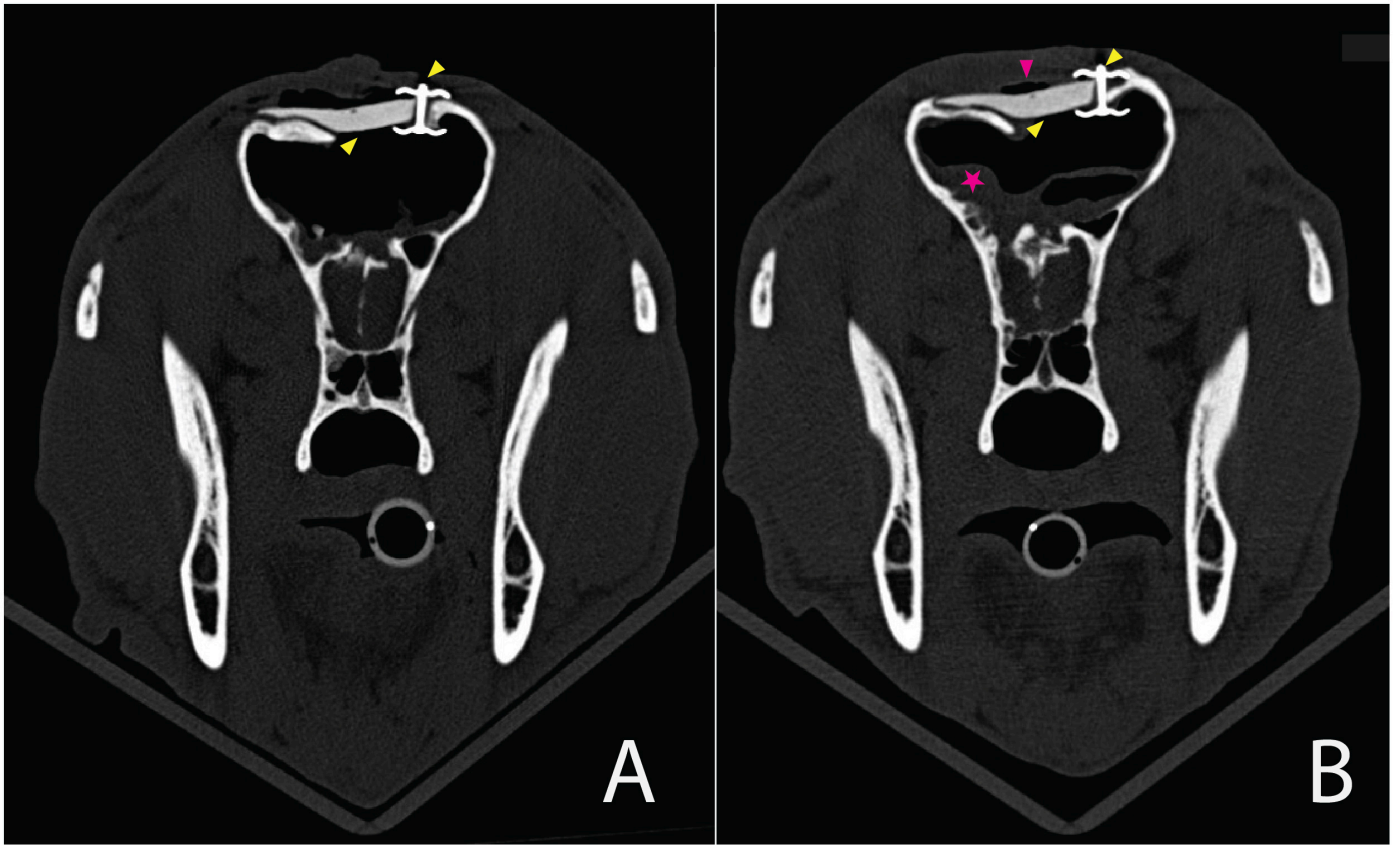
Transverse views in bone window at the level of the mandibular rami and frontal sinus. **(A)** Immediately postsurgical after PMMA placement, the PMMA plate and metallic “umbrella” device (ag Aesculap^®^ CranioFix^®^2 system) marked with yellow arrows. **(B)** Four months after debridement, the implants appear unchanged in position. No osteolysis apparent surrounding the implants. A small amount of fluid is seen within the reconstructed frontal sinus (pink asterisk) together with a small volume of subcutaneous gas dorsal to the plate (pink arrow).

**Figure 6:**
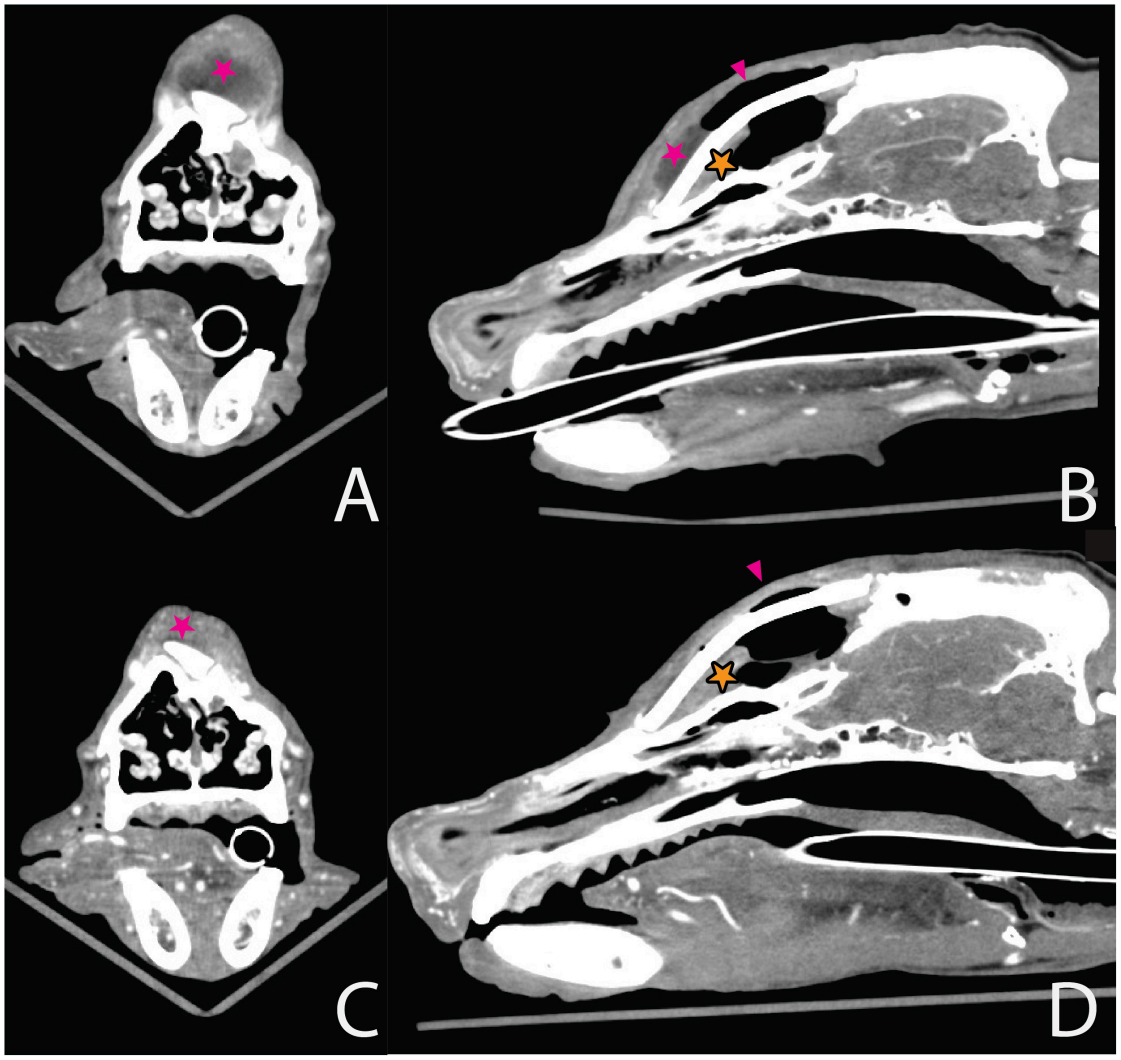
Transverse post-contrast views at the level of the nasal bridge and rostral part of the plate **(A, C)** and mid-sagittal views in soft tissue window **(B, D)**. **(A, B)** 2 weeks after presentation with local swelling and draining tract over the nasal bridge–local soft tissue swelling with peripherally contrast-enhanced rim and a fluid attenuated center (pink asterisk). **(C, D)** Four months later, in the same location, there was no indication of previous findings. A small amount of non-enhancing fluid within the reconstructed frontal sinus of the same volume (orange asterisk).

Immediately following the surgery, a CT scan was performed to evaluate the plate placement. The plate was positioned over the forehead overlaying the caudal part of the nasal bone rostrally and extending caudally over the frontal bones, bridging the gap over the frontal sinuses. Closer contact between the frontal bone and the overlaying plate was achieved caudally, where a titanium fixation clamp anchored the plate to the left frontal bone. Multiple hyperattenuating antibiotic beads placed in the sinus cavity during surgery were demonstrated. The dog was hospitalized in the ICU for 1 day and was then discharged back to its unit with clindamycin 12.5 mg/kg, PO, q12h for 10 days; gabapentin 10 mg/kg, PO, q12h for 7 days, dipyrone 25 mg/kg, PO, q12h for 3 days, and instructions for rest. The dog presented for planned follow-up examinations 7 and 14 days after the second surgery. At the 7-day follow-up, the handler reported no concerns; the dog was alert, active, and behaving normally. Physical examination was normal except for mild swelling and subtle pain around the surgical area, but no plate mobility was detected on palpation. At the 14-day follow-up, the wound was completely healed and sutures were removed. There was no pain or plate mobility upon facial palpation. The handler was very satisfied with the esthetic outcome, as the dog had a symmetric facial appearance. The dog received clindamycin for 1 month without any adverse effects. Thirteen months after surgery, there had been no evidence of infection or other complications. On physical examination there were no draining tracts, no local swelling/erythema or temperature increase, no pain on palpation, no nasal discharge, and no detectable implant mobility or pain on firm bilateral palpation of the surgical area. Fourteen months after surgery, the dog represented with focal swelling and a draining tract over the nasal bridge. The referring veterinarian suspected trauma from a rigid metal muzzle, and antibiotics were initiated. CT performed ~2 weeks later demonstrated that the PMMA plate and the metallic agAesculap^®^ CranioFix^®^2 system remained stable in position relative to the surrounding bones. Mild bone remodeling was noted, but no lytic changes were present around the implant. A small to moderate amount of non-enhancing content was observed in the ventral reconstructed frontal sinuses, likely representing exudative fluid and secretions. Subcutaneous gas was evident dorsal to the plate and ventrally within the sinuses and nasal passages. In addition, soft tissue swelling was present dorsal to the nasal bridge, with a peripherally contrast-enhanced rim and fluid-attenuating center, findings compatible with an abscess or localized infection. No definite fistulous tract was identified (Figures 5A, 6A, B). Surgical debridement and irrigation were performed. No tracts were observed connecting the draining tract and abscess with the sinus or plate. Tissue culture grew *Staphylococcus pseudintermedius*, susceptible to fluoroquinolones, gentamicin, sulfamethoxazole/trimethoprim, penicillinase-resistant penicillins, and first-generation cephalosporins. The wound was closed with a drain, which was removed after 3 days. Clinically, the dog improved markedly on enrofloxacin, with a substantial reduction in discharge.

A repeat CT, performed 4 months after the debridement, showed mild thickening of the soft tissue over the plate but no fluid accumulation. A small amount of gas persisted at the level of the frontal sinuses. Taken together, implant stability, lack of periosteal reaction, an aerated sinus with only a minor non-enhancing pocket, and absence of a sinus-directed tract at surgery, these findings do not support the spread of infection from the frontal sinus (Figures 5B, 6C, D).

## Discussion

To date, no guidelines exist for managing frontal sinus fractures in dogs. This treatment was adapted from human medical protocols and general fracture management principles. Human fractures are classified into anterior and posterior wall fractures, with or without displacement and CSF leaks (5). Posterior fractures necessitate surgical intervention to restore the barrier between the frontal sinus and the cerebral cavity (6). The treatment for anterior fractures depends on the degree of fragment displacement and sinonasal obliteration.

In this case, the dog presented with an open, contaminated anterior fracture and damage to upper respiratory pathways, evident on initial physical examination. A CT scan was the imaging modality chosen in order to evaluate the trauma, as it provides optimal visualization of bone and soft tissue defects and allows rapid identification of foreign bodies and intracranial hemorrhage, enabling precise surgical planning. CT is considered the gold standard for trauma imaging of the CMF region (1–4). The CT also revealed multiple cribriform plate fractures resulting in a communication between the frontal sinus and the intracranial cavity and consequential mild pneumocephalus. Magnetic resonance imaging (MRI) was not performed, as the primary focus was on evaluating bone and upper airway damage. The dog's near-normal neurological status made further cerebral imaging unnecessary, although MRI could be beneficial in similar cases to assess brain injury more comprehensively. The CT identified several issues requiring intervention: open contaminant fractures of the frontal and nasal bones, and communication between the frontal sinus, skull cavity, and brain. Based on human medicine, where nasofrontal duct obstruction is managed through sinus obliteration (11), we decided to occlude the frontal sinus airflow. A temporary tracheostomy was performed to bypass the upper respiratory tract, reduce pressure on the brain, and prevent pneumocephalus progression. The tracheostomy was maintained for 6 days to facilitate closure of the pathological connections.

Pneumocephalus refers to gas in the cranial cavity, often occurring after skull fractures or surgeries. In humans, tension pneumocephalus can lead to increased intracranial pressure and neurological deficits, but spontaneous resolution is common (12). Tension pneumocephalus is significant as it can lead to increased intracranial pressure and progressive neurological deficits. In humans, tension pneumocephalus is predominantly subdural and is commonly associated with trauma, skull base tumors, and, less frequently, craniotomy or otorhinolaryngological procedures. Spontaneous cases have also been reported (13). Intraventricular tension pneumocephalus in humans is relatively rare and typically occurs as a complication of ventriculoperitoneal shunting (14). In contrast, all reported cases of tension pneumocephalus in dogs have been primarily intraventricular, accompanied by craniocervical subarachnoid gas accumulation, mostly following transfrontal craniectomy/craniotomy for brain tumor removal (15–18), dorsal rhinotomy with cribriform plate damage (19–21), and trauma with frontal bone fractures (22, 23). Neurological deterioration associated with tension pneumocephalus has been reported in a delayed timeframe, ranging from 1 week to 5 months following the initial procedure. In cases of tension pneumocephalus with traumatic etiology, neurological deterioration has been reported to occur as early as 3 weeks in one case and as late as 15 months in another (22, 23). In both cases, the neurological deterioration was the indication for surgical repair of the dural defect. This was accomplished either by using a synthetic dural substitute or a fascial graft, to improve the barrier between the intracranial compartment and the frontal sinus. While it was not tension pneumocephalus in our case, monitoring it remained important as it indicated the integrity of the barrier between the sinus and cranial cavity. The absence of new pneumocephalus on follow-up CT scans at 3 and 8 weeks, and 14 months along with a stable neurological status, indicated successful repair. In humans, a trial study on traumatic pneumocephalus associated with mild to moderate head trauma found that ~20% of patients developed septic meningoencephalitis. Independent risk factors identified in that study were the presence of CSF rhinorrhea and intracranial hemorrhage (24). Due to massive trauma to the nasal cavity in our case, it was difficult to evaluate the absence of CSF rhinorrhea, and there was evidence of minor intracranial hemorrhage. Considering these factors, we found it critically important to prevent the development of an infection in the frontal sinus/cranium cavity and the onset of meningoencephalitis. To achieve this, we formed a barrier between the frontal sinus cavity and the cranial cavity; treated the open fracture of the frontal sinus; and achieved final closure of the frontal sinus.

In human medicine, bone grafts are typically used for skull defects larger than 30 mm to provide structural support (25, 26). For dogs, a technique using temporalis fascia and fat grafts has been described for dural defect closure (15, 27). In our case, synthetic materials were used for the dural closure due to significant tissue loss and contamination.

Open fractures are classified by the Gustilo-Anderson system (28). In our case, type III fractures involving extensive soft tissue damage and a high risk of infection. Bacterial colonization can lead to osteomyelitis if not managed with appropriate debridement and antimicrobial therapy. In human medicine, numerous advantages of using antibiotic-impregnated beads over systemic therapy have been recognized (29). We placed antibiotic-impregnated beads in the frontal sinus to maintain high local concentrations of antibiotics, alongside systemic antibiotics until culture results were available (30). With negative results, systemic antibiotics were discontinued, and no infection was noted in a follow-up CT scan 3 weeks after the first surgery. This allowed us to evaluate the size and shape of the external frontal bone defect, which assessed our decision-making regarding the method for the final reconstructive surgery.

The decision to employ a two-stage surgical approach was primarily guided by infection control considerations. Given the complexity of the case and the substantial risk of postoperative infection, it was determined that a staged strategy would optimize patient outcomes by allowing adequate wound healing and infection monitoring before definitive reconstruction. While acknowledging that PMMA offers excellent malleability, several factors influenced the decision regarding its immediate use. First, the characteristics of the defect: the extensive bone loss in this case would have necessitated a large PMMA reconstruction. Fabricating an individualized prosthesis of such magnitude during the initial procedure posed challenges in achieving precise contouring while maintaining surgical efficiency. Second, the quality of reconstruction: although PMMA can be shaped subjectively to fit the defect, achieving an anatomically accurate reconstruction requires careful attention to surface smoothness and appropriate thickness to minimize complications such as pressure necrosis or prosthesis exposure. The two-stage approach is supported by veterinary literature describing similar cases. Bryant et al. (8) reported cranioplasty procedures using individually molded PMMA prostheses in two dogs following tumor excision. Their technique involved creating sterile aluminum foil molds to achieve custom-fitted reconstructions. However, their case series also highlighted potential complications associated with immediate PMMA placement. One patient developed a fistulous tract 1 month postoperatively, with visible PMMA exposure through the medial canthus. The authors attributed this complication to suboptimal prosthesis contouring, particularly at the edges of the dorsal orbital rims, where pressure necrosis of adjacent tissue facilitated bacterial invasion. Their study emphasized that prosthetic reconstruction presents inherent challenges in distributing pressure evenly across overlying tissues while avoiding distortion of the palpebral fissure or interference with eyelid function. Individualized surgical planning that considers patient-specific factors and defect characteristics is therefore essential to achieving optimal outcomes in these complex cases.

Reconstructive surgery aims for biomechanical stability, cerebral protection, and cosmetic restoration (10, 11). When the frontal sinus is involved, the general recommendation in humane medicine is to avoid incomplete sinus and nasal occlusion that can lead to abnormal communication between the microbiome of the sinus/nasal cavity and the subcutaneous tissue, resulting in abscess, pyocele, chronic rhinitis, or sinusitis. To meet these goals, we used a 3D PMMA implant. We choose PMMA over metal due to several limitations of metallic implants: difficulty in shaping, heat and electrical conductivity, and radiopacity. Additionally, a complete metal implant will be heavy, although there are descriptions of frontal sinus reconstruction using titanium miniplates and a lighter and more flexible titanium mesh (31). PMMA offers several useful features: biocompatibility, stability *in vivo*, and absence of the inherent metal. It is much lighter yet strong, with tensile and flexural strength greater than that of bone. Furthermore, it is radiolucent and does not produce artifacts in advanced imaging (CT/MRI), which can be crucial in future evaluation of the patient. The biomechanical properties of PMMA for cranioplasty have been well studied in humans, both *in vitro* and in clinical cases (31, 32). This has made it the most widely used synthetic material for this type of surgery, alongside bone grafts (31). One, significant disadvantage of PMMA is the high temperature generated during its exothermic reaction while stabilization (reaching 70 °C−80 °C within 15 min after mixing). This can lead to necrosis of the surrounding tissue. Although constant irrigation with cold saline solution is recommended to prevent thermal injury, this method is not always effective and it is impossible to evaluate the extent of damage immediately during the procedure. This risk can be avoided by preparing the implant before placement, a technique described in human medicine since the 1980s. This approach has evolved significantly from using an impression of the head defect and creating a positive cast in dental stone (32) to modern techniques utilizing patient-specific 3D-printed implants (33). In the veterinary field, only three papers describing cranioplasty using PMMA in dogs were published, covering four cases, all following skull tumor removal, with three cases involving the frontal sinus (8, 34, 35). In the present case, we decided to use an individualized patient-specific 3D implant, made based on post-surgery CT imaging, to avoid geometric mismatches between the implant and the bone defect and residual soft tissue volume. To minimize pressure on subcutaneous tissue and skin, we worked in close collaboration with the Customized Medical Devices company to develop the shape of the implant. The design allowed the implant to partially sink into the frontal sinus, with its upper surface coinciding with the surface of the surrounding skull bones, thus minimizing pressure on bones and soft tissues. Immediately after surgery, a control CT was performed to evaluate the successful placement of the implant. Thirteen months after surgery, there had been no evidence of infection or other complications. Fourteen months after surgery, the dog developed focal swelling and a draining tract, suspected to be related to external trauma from a metal muzzle. Imaging, surgical exploration, and culture of Staphylococcus pseudintermedius were more consistent with a localized soft-tissue infection than with implant- or sinus-associated disease. While trauma appears to be the most likely explanation, repeat CT after debridement and antibiotic therapy demonstrated implant stability and resolution of fluid accumulation, Assessment included superficial, trauma-associated problem, likely exacerbated by the limited soft-tissue thickness between skin, nasal bone, and the cranial part of the plate or intermittent contact/communication between the cranial plate margin and the sinonasal space as a potential route of contamination. In human medicine, CT follow-up is routinely performed; however, direct comparisons to dogs are limited by major anatomical differences. The human frontal sinus is much smaller, and when involved in craniotomy, surgical techniques often employ complete filling of the sinus with PMMA. In dogs, such an approach would require a substantially larger volume of PMMA, potentially generating an exothermic reaction that could theoretically endanger the brain, a complication not reported to date (36). Moreover, large clinical series describing surgical site infection (SSI) rates in people primarily involve planned craniotomies (37), which may not be directly comparable to our case. In veterinary medicine, late CT surveillance is generally not performed unless clinically indicated, due to the need for general anesthesia. To our knowledge, only a single report has described long-term CT-confirmed outcomes following PMMA cranioplasty in dogs, performed because of recurrent clinical signs. In that study, one dog remained clinically stable 3 years postoperatively without infection or implant-related complications, whereas another developed SSI that ultimately required implant removal (8).

## Conclusions

This case highlights the successful management of complex open craniomaxillofacial trauma with brain involvement. A two-stage approach initially securing cranial cavity integrity and addressing infection, followed by placement of an individualized 3D PMMA implant, resulted in excellent functional and cosmetic outcomes. Both short-term and long-term evaluations showed no major complications, demonstrating the success of this novel treatment strategy.

## Data Availability

The original contributions presented in the study are included in the article/supplementary material, further inquiries can be directed to the corresponding author.

## Footnotes

1 Advia 2120i, Siemens Medical Solutions Diagnostics GmbH, Erfurt, Germany.

2 Cobas Integra 400 Plus, Roche, Mannheim, Germany.

3 Septal scrub, Vitamed pharmaceutical industries ltd, Bimiamina, Israel.

4 Sodium chloride, 0.9%, Baxter healthcare Ltd, Teva medical, Ashdod, Israel.

5 Fentanyl—Kalceks 0.05 mg/ml, HBM Pharma s.r.o. Sklabinska, Slovakia.

6 Lactate ringer solution, hartman, Baxter Healthcare L.t.d, Thetford, UK.

7 Clindamycin 150 mg/ml injection. Manufacturer: Rafa Laboratories Ltd., Jerusalem, Israel.

8 Midazolam hydrochloride, Kalceks, Riga, Latvia.

9 Propofol 1% MCT Fresenius, Cure Medical and Technical supply, Petach-Tikva, Israel.

10 PraminS.A.L.F.S.p.A, Cenate, Sotto, Italy.

11 Lidocaine hydrochloride S.A.L.F., RAZ Pharmaceutics, Haderha, Israel.

12 Ketamin, PRODULAB PHARMA B.V., Holland.

13 Baytril 5%, BAYER Animal Health GMBH, Leverkusen, Germany.

14 Keppra, UCB Pharma, Brussels, Belgium.

15 CERENIA^®^, maropitant, citrate injection, Zoetis Inc. Kalamazoo, Ireland.

16 Ulceron, Anfarm Hellas S.A, Athens, Greece.

17 Trazadone, UNIPHARM LTD, Mevo Carmel, Israel.

18 Diazepam, Assival, Teva medical, Petch-Tikve, Israel.

19 Isuflorane, Primal Critical Care, Inc. Bethlehem, USA.

20 Oxidized regenerated Celluloseu (Equitamp^®^), Equimedical, Zwaneburg, the Netherlands.

21 Dural Regeneration Matrix DuraGen Secure, Integra LifeSciences Corporation, Princeton, USA.

22 Stratasys J55 printer with Med610 material from Stratasys, manufactured by Synergy3DMed, Netanya, Israel.

23 Equitamp^®^, Equimedical BV, Zwaneburg, Netherlands.

24 Cellulose sponges, Ethicon SARL, Neuchatel, Switzerland.

25 Polydioxanone, PDO absorbable monofinament suture 3-0, AssuCrylMonoSlow, Assut Sutures, Switzerland.

26 Polyamide, Nylon non-absorbable monofinament suture 3-0, AssuCrylMonoSlow, Assut Sutures, Switzerland.

27 Clindamycin, Dalacin, FAREVA AMBOISE, Pocé-sur-Cisse, France.

28 Ofloxacin, Oflodex, Dexel, Or-Akiva, Israel.

29 Dypiron, Optalgin, Teva Medical, Petach-Tikva, Israel.

30 Mimics Software, Materialise, Belgium.

31 CAD software. FDA approved, 3Matics by Materilise, Belgium.

32 Biomed Clear by formlabs, USA.

33 Methadone, RAFA, Jeruslem, Israel.

34 Acepromazine, Vetoquinol, France.

35 GLUture Topical Tissue Adhesive, Zoetis, New Jersey, USA.

36 Aesculap^®^ CranioFix^®^2 system, Aesculap, Tuttlingen, Germany.

## References

[1] De Paolo MH Arzi B Pollard RE Kass PH Verstraete FJM. Craniomaxillofacial trauma in dogs—part I: fracture location, morphology and etiology. Front Vet Sci. (2020) 7:241. doi: 10.3389/fvets.2020.00241 32411743 PMC7199291

[2] De Paolo MH Arzi B Pollard RE Kass PH Verstraete FJM. Craniomaxillofacial trauma in dogs—part II: association between fracture location, morphology and etiology. Front Vet Sci. (2020) 7:242. doi: 10.3389/fvets.2020.00242 32478108 PMC7242568

[3] Amengual-Batle P José-López R Durand A Czopowicz M Beltran E Guevar J . Traumatic skull fractures in dogs and cats: a comparative analysis of neurological and computed tomographic features. J Vet Int Med. (2020) 34:1975–85. doi: 10.1111/jvim.15838 32686202 PMC7517851

[4] Wolfs E Arzi B Cota JG Kass PH Verstraete FJM. Craniomaxillofacial trauma in immature dogs–etiology, treatments, and outcomes. Front Vet Sci. (2022) 9:932587. doi: 10.3389/fvets.2022.932587 36090162 PMC9449964

[5] Schütz P Ibrahim HHH Rajab B. A Textbook of Advanced Oral and Maxillofacial Surgery Volume 2. Motamedi MHK, editor. London: IntechOpen (2015). doi: 10.5772/59096

[6] Kim YW Lee DH Cheon YW. Secondary reconstruction of frontal sinus fracture. Arch Craniofac Surg. (2016) 17:103–10. doi: 10.7181/acfs.2016.17.3.103 28913266 PMC5556797

[7] Dunand L Belluzzi E Bongartz A Caraty J. Application of a bilateral temporal fascia free graft in a dog with multifragmented frontal sinus and nasal bone fracture. Vet Surg. (2022) 51:1002–8. doi: 10.1111/vsu.13804 35289944

[8] Bryant KJ Steinberg H McAnulty JF. Cranioplasty by means of molded polymethylmethacrylate prosthetic reconstruction after radical excision of neoplasms of the skull in two dogs. J Am Vet Med Assoc. (2003) 223:67–72. doi: 10.2460/javma.2003.223.67 12839066

[9] Langer P Black C Egan P Fitzpatrick N. Treatment of calvarial defects by resorbable and non-resorbable sonic activated polymer pins and mouldable titanium mesh in two dogs: a case report. BMC Vet Res. (2018) 14:199. doi: 10.1186/s12917-018-1506-0 29929513 PMC6013898

[10] Arzi B Verstraete FJM. Internal fixation of severe maxillofacial fractures in dogs. Vet Surg. (2014) 44:437–42. doi: 10.1111/j.1532-950X.2014.12161.x 24512370

[11] Rohrich RJ Hollier LH. Management of frontal sinus fractures. Changing concepts. Clin Plast Surg. (1992) 19:219–32. doi: 10.1016/S0094-1298(20)30905-6 1537220

[12] Reasoner DK Todd MM Scamman FL Warner DS. The incidence of pneumocephalus after supratentorial craniotomy. Anesthesiology. (1994) 80:1008–12. doi: 10.1097/00000542-199405000-00009 8017640

[13] Schirmer CM Heilman CB Bhardwaj A. Pneumocephalus: case illustrations and review. Neurocrit Care. (2010) 13:152–8. doi: 10.1007/s12028-010-9363-0 20405340

[14] Salem-Memou S Vallee B Jacquesson T Jouanneau E Berhouma M. Pathogenesis of delayed tension intraventricular pneumocephalus in shunted patient: possible role of nocturnal positive pressure ventilation. World Neurosurg. (2015) 85:365.e17–20. doi: 10.1016/j.wneu.2015.09.001 26363220

[15] Cavanaugh RP Aiken SW Schatzberg SJ. Intraventricular tension pneumocephalus and cervical subarachnoid pneumorrhachis in a bull mastiff dog after craniotomy. J Small Anim Pract. (2008) 49:244–8. doi: 10.1111/j.1748-5827.2007.00467.x 18373545

[16] Garosi LS Penderis J Brearley MJ Brearley JC Dennis R Kirkpatrick PJ. Intraventricular tension pneumocephalus as a complication of transfrontal craniectomy: a case report. Vet Surg. (2002) 31:226–31. doi: 10.1053/jvet.2002.32449 11994849

[17] Garosi LS McConnell FJ Lujan A. What is your diagnosis? J Am Vet Med Assoc. (2005) 226:1057–8. doi: 10.2460/javma.2005.226.1057 15825729

[18] Hicks J Stewart G Kent M Platt S. Delayed asymptomatic progressive intraventricular pneumocephalus in a dog following craniotomy. J Small Anim Pract. (2018) 61:316–20. doi: 10.1111/jsap.12858 29736904

[19] Fletcher DJ Snyder JM Messinger JS Chiu AG Vite CH. Ventricular pneumocephalus and septic meningoencephalitis secondary to dorsal rhinotomy and nasal polypectomy in a dog. J Am Vet Med Assoc. (2006) 229:240–5. doi: 10.2460/javma.229.2.240 16842045

[20] Heading KL Nicoll RG Bennett PF. Intra-ventricular pneumocephalus and intra-nasal meningoencephalocele following dorsal rhinotomy. Aust Vet Pract. (2011) 41:133–5.

[21] Launcelott ZA Palmisano MP Stefanacci JD Whitney BL. Ventricular pneumocephalus, cervical subarachnoid pneumorrhachis, and meningoencephalitis in a dog following rhinotomy for chronic fungal rhinitis. J Am Vet Med Assoc. (2016) 248:430–5. doi: 10.2460/javma.248.4.430 26829276

[22] Haley AC Abramson C. Traumatic pneumocephalus in a dog. J Am Vet Med Assoc. (2009) 234:1295–8. doi: 10.2460/javma.234.10.1295 19442025

[23] Thieman K Echandi RL Arendse A Henry G. Imaging diagnosis—trauma-induced tension pneumocephalus. Vet Radiol Ultrasound. (2008) 49:362–4. doi: 10.1111/j.1740-8261.2008.00381.x 18720768

[24] Eftekhar B Ghodsi M Nejat F Ketabchi E Esmaeeli B. Prophylactic administration of ceftriaxone for the prevention of meningitis after traumatic pneumocephalus: results of a clinical trial. J Neurosurg. (2004) 101:757–61. doi: 10.3171/jns.2004.101.5.0757 15540912

[25] Church CA Chiu AG Vaughan WC. Endoscopic repair of large skull base defects after powered sinus surgery. Otolaryngology. (2003) 129:204–9. doi: 10.1016/S0194-5998(03)00521-7 12958568

[26] Rodrigues M O'Malley BW Staecker H Tamargomd R. Extended pericranial flap and bone graft reconstruction in anterior skull base surgery. Otolaryngology. (2004) 131:69–76. doi: 10.1016/j.otohns.2004.02.033 15243560

[27] Kostolich M Dulisch ML. A surgical approach to the canine olfactory bulb for meningioma removal. Vet Sur. (1987) 16:273–7. doi: 10.1111/j.1532-950X.1987.tb00952.x 3507155

[28] Zimmerli W editor. Bone and Joint Infections: From Microbiology to Diagnostics and Treatment. Hoboken, NJ: John Wiley & Sons, Inc (2021). doi: 10.1002/9781119720676

[29] Gogia J Meehan J Di Cesare P Jamali A. Local antibiotic therapy in osteomyelitis. Semin Plast Surg. (2009) 23:100–7. doi: 10.1055/s-0029-1214162 20567732 PMC2884906

[30] Gieling F Peters S Erichsen C Richards RG Zeiter S Moriarty TF. Bacterial osteomyelitis in veterinary orthopaedics: pathophysiology, clinical presentation and advances in treatment across multiple species. Vet J. (2019) 250:44–54. doi: 10.1016/j.tvjl.2019.06.003 31383419

[31] Park HK Dujovny M Agner C Diaz FG. Biomechanical properties of calvarium prosthesis. Neurol Res. (2001) 23:267–76. doi: 10.1179/016164101101198424 11320607

[32] Van Gool AV. Preformedpolymethylmethacrylate cranioplasties: report of 45 cases. J Maxillofac Surg. (1985) 13:2–8. doi: 10.1016/S0301-0503(85)80005-9 3856619

[33] Czyżewski W Jachimczyk J Hoffman Z Szymoniuk M Litak J Maciejewski M . Low-cost cranioplasty—a systematic review of 3D printing in medicine. Materials. (2022) 15:4731. doi: 10.3390/ma15144731 35888198 PMC9315853

[34] Moissonnier P Devauchelle P Delisle F. Cranioplasty after en bloc resection of calvarial chondroma rodens in two dogs. J Small Anim Pract. (1997) 38:358–63. doi: 10.1111/j.1748-5827.1997.tb03486.x 9282343

[35] Chitty JW Miro AC Kaczmarska A Guevar J Gutierrez-Quintana R. Frontal sinus repair using polymethyl methacrylate after craniectomy for a resection of a fronto-parietal osteoma in a dog. Vet Rec Case Rep. (2021) 9:e209. doi: 10.1002/vrc2.209

[36] Matsuura J Otsuka T Nakagawa T Kai K. Frontal sinus repair using polymethyl methacrylate after bifrontal craniotomy. World Neurosurg. (2019) 124:e281–4. doi: 10.1016/j.wneu.2018.12.083 30593968

[37] Kim MJ Lee HB Ha SK Lim DJ Kim SD. Predictive factors of surgical site infection following cranioplasty: a study including 3D printed implants. Front Neurol. (2021) 12:745575. doi: 10.3389/fneur.2021.745575 34795630 PMC8592932

